# *In Situ* Genetic Evaluation of European Larch Across Climatic Regions Using Marker-Based Pedigree Reconstruction

**DOI:** 10.3389/fgene.2020.00028

**Published:** 2020-02-13

**Authors:** Milan Lstibůrek, Silvio Schueler, Yousry A. El-Kassaby, Gary R. Hodge, Jan Stejskal, Jičí Korecký, Petr Škorpík, Heino Konrad, Thomas Geburek

**Affiliations:** ^1^ Faculty of Forestry and Wood Sciences, Czech University of Life Sciences, Praha, Czechia; ^2^ Department of Forest Growth and Silviculture, Federal Research and Training Centre for Forests, Natural Hazards and Landscape (BFW), Wien, Austria; ^3^ Department of Forest and Conservation Sciences, Faculty of Forestry, University of British Columbia, Vancouver, BC, Canada; ^4^ Department of Forestry and Environmental Resources, North Carolina State University, Raleigh, NC, United States; ^5^ Department of Forest Genetics, Federal Research and Training Centre for Forests, Natural Hazards and Landscape (BFW), Wien, Austria

**Keywords:** genetic evaluation, pedigree reconstruction, sustainable forestry, European larch, genetic gain, forest tree breeding

## Abstract

Sustainable and efficient forestry in a rapidly changing climate is a daunting task. The sessile nature of trees makes adaptation to climate change challenging; thereby, ecological services and economic potential are under risk. Current long-term and costly gene resources management practices have been primarily directed at a few economically important species and are confined to defined ecological boundaries. Here, we present a novel *in situ* gene-resource management approach that conserves forest biodiversity and improves productivity and adaptation through utilizing basic forest regeneration installations located across a wide range of environments without reliance on structured tree breeding/conservation methods. We utilized 4,267 25- to 35-year-old European larch trees growing in 21 reforestation installations across four distinct climatic regions in Austria. With the aid of marker-based pedigree reconstruction, we applied multi-trait, multi-site quantitative genetic analyses that enabled the identification of broadly adapted and productive individuals. Height and wood density, proxies to fitness and productivity, yielded *in situ* heritability estimates of 0.23 ± 0.07 and 0.30 ± 0.07, values similar to those from traditional “structured” pedigrees methods. In addition, individual trees selected with this approach are expected to yield genetic response of 1.1 and 0.7 standard deviations for fitness and productivity attributes, respectively, and be broadly adapted to a range of climatic conditions. Genetic evaluation across broad climatic gradients permitted the delineation of suitable reforestation areas under current and future climates. This simple and resource-efficient management of gene resources is applicable to most tree species.

## Introduction

The composition, function, and service of terrestrial ecosystems are increasingly threatened by the steady global warming trend (Walther et al., 2002; Walther, 2010; Hanewinkel et al., 2013). Plants' immediate response to climate change is manifested in altered phenology (Wolkovich et al., 2012), increased growth (Pretzsch et al., 2014), and mortality (Allen et al., 2010). Assisted gene flow has been considered as a viable option for dealing with the mismatch between environmental alterations caused by climate change and the migration pace of plant populations (McLachlan et al., 2007; Kremer et al., 2012). However, assisted gene flow has not been thoroughly tested as genotypes are transferred to novel environments with altered thermal (Vitt et al., 2010), photoperiod (Saikkonen et al., 2012; Frascaria-Lacoste and Fernández-Manjarrés, 2012), and edaphic conditions (Kranabetter et al., 2012). Furthermore, epigenetic after-effects associated with plants transfer (Holeski et al., 2012; Bräutigam et al., 2013) and phenotypic plasticity (Alberto et al., 2013) have been discounted. These factors, collectively, provide sound reasons to explore alternative forest tree gene management approaches.

Forest tree gene resource management, with concurrent selective breeding and gene conservation, are long-term endeavors involving hundreds of parents and thousands of offspring tested at multiple locations, requiring substantial resources, elaborate logistics, and sustained organizational commitment, and more importantly, are predominantly encapsulated within specific ecological boundaries known as breeding zones (White et al., 2007). These extensive programs often follow the recurrent selection scheme with repeated rounds of breeding, testing, and selection, resulting in cumulative improvement (genetic response to selection) delivered through specialized seed production populations known as seed orchards. In conventional selective breeding programs, controlled pollinations following specific mating designs produce structured pedigrees (White et al., 2007), which are evaluated in replicated test sites within defined ecological boundaries (Hanewinkel et al., 2013), a prerequisite for effective genetic evaluation and selection. These considerable efforts are restricted to few economically important species, thus facilitating widespread cultivation of few species, with potential adverse effects on tree species diversity and ecological services provision (Isbell et al., 2015; Hua et al., 2016). Moreover, reforestation with orchard-produced seedlings is restricted to their respective ecological boundaries; thus, these programs can be spatially static and slow in responding to environmental contingencies or market demands.

European larch, an economically important deciduous conifer, is distributed in Central Europe. It is native to the Alps and the Carpathian Mountains, with smaller disjunct populations in northeastern Europe. This shade-intolerant species is primarily planted within mixed forests due to its high ecological value and excellent wood characteristics. Despite its wide planting outside of its native range, the gene resource management effort for the species is mainly focused on seed provision and gene conservation (Pâques et al., 2013). The species occurs naturally across a discontinuous range in the Alps, Sudetes, and Carpathians, as well as in Polish lowlands. This shade-intolerant species shows a subcontinental climate preference and high site tolerance. Due to its high resistance and durability, larch wood is a traditional material for building and roof construction in the Alpine area, with increasing importance in modern architecture and furniture design. As *L. decidua* has the finest wood characteristics among temperate European conifers, it has been widely planted throughout the continent in artificial plantations, thus facilitating translocations of genetic materials for more than three centuries (Jansen and Geburek, 2016). Under climate change conditions, the species appeared to be highly vulnerable to drought events (Allen et al., 2010). *L. decidua* shows high levels of genetic variation including drought sensitivity across the species range (George et al., 2017). Thus, the species regional improvement activities are focused on conserving its genetic diversity and utilizing local sources for increasing the species adaptation to climate change.

At the northeastern fringe of the Alps, European larch improvement activities are conducted within a spatially and climatically heterogeneous landscape. This region reaches from lowland areas around the river Danube (∼200 m a.s.l.) through the hilly landscape of the alpine foreland up to mountains of 900 m in the northern calcareous Alps. Present climate conditions are represented by four climatic zones: 1) pannonical continental climate with hot summers and frequent droughts in low elevations of the East and Northeast, 2) temperate Atlantic climate with warm temperatures and frequent precipitations in the western Alpine foreland, 3) temperate climate with continental influence at low elevations of the Eastern Alps, and 4) harsh mountain conditions at higher elevation with lower temperatures and low winter temperatures. Global warming in the Alpine region has already resulted in a significant 2)∘C temperature increase since 1880, about twice as high as the global average (Allen et al., 2010).

Utilizing the European larch breeding program in Austria, we investigated a feasible alternative that would efficiently address the global climate change issues and overcome the major limitations of assisted gene flow, and deliver substantial production and social benefits to the human society. We analyzed 25- to 35-year-old larch progenies originating from open pollination in a common parental source (a seed orchard) and growing in 21 reforestation installations (typical forest stands). Utilizing the “Breeding-without-Breeding” methodology with phenotypic preselection (El-Kassaby and Lstibůrek, 2009; Lstibůrek et al., 2015), we reconstructed the parentage of individual progenies and estimated heritabilities for height and wood density yielding similar values to those from typical full-sib forest genetic trials. Genetic evaluation across broad climatic gradients permitted the delineation of suitable reforestation areas under current and future climates. Following the evaluation, the second-generation seed orchard was established from the top-ranking selections.

## Materials and Methods

### Source Population and Climate Data

The seed orchard [Nat. Reg. No. Lä P3 (4.2/sm-tm)] for which we aimed to conduct accelerated gene-resource management is located at an altitude of 520 m a.s.l., and its seed material is considered to be the most valuable larch seeds for the mountainous areas of the northern alpine foreland. The orchard was established in 1954 over 3.15 ha, with 1,666 vegetative propagules of 53 phenotypically selected parent trees. Since its establishment, the main objective of the Austrian larch seed orchard program was to secure seed supply with minimal genetic testing; thus, controlled pollinations and progeny tests were not conducted.

Next, we identified 21 reforestation installations (sites) within comparable tree ages (25 to 37 years), sufficient size (at least 200 remaining trees in more or less regular planting designs), low level of environmental variation within the site, and composition in which larch is the single or dominant tree species. These 21 reforestation installations all originated from mixed seedlots harvested from the seed orchard, are located at altitudes between 250 and 800 m, and span through a geographical space of about 170 km W-E and 110 km N-S.

**Figure 1:**
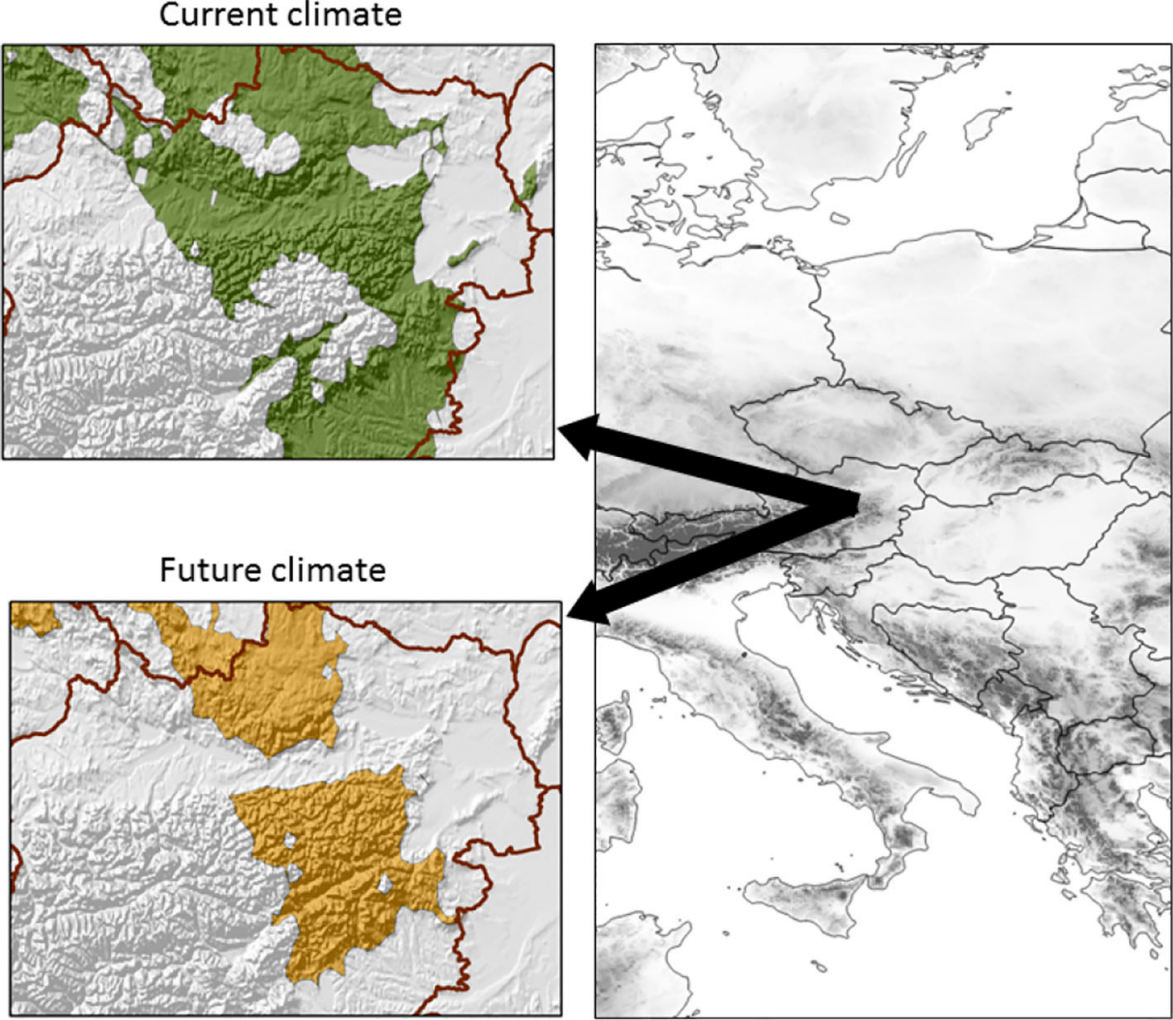
Potential planting area of selected superior larch genotypes under current (top) and expected future (2050) climate conditions (bottom) at the northwestern fringe of the Alpine area.

Climate data for the 21 reforestation installations with putative seed deployment areas under the present and future climate conditions were obtained from the locally downscaled high-resolution WorldClim models (Hijmans et al., 2005); WorldClim has a spatial resolution of 30 arc-seconds. For an unbiased climate comparison, we obtained all monthly climate parameters (average monthly mean, minimum and maximum temperatures, average monthly precipitation) as well as various derived bioclimatic variables (Meier et al., 2012), and the impact of these climate descriptors, as well as their inter-correlations, was distilled through principal component analysis (PCA) in which the climate of the reforestation sites was used as active cases. Potential/future seed deployment areas in Austria were identified by including gridded climate data as inactive cases (Meier et al., 2012). As these deployment areas should cover the broad range of the four climatic groups, we used the maximum and minimum of the 21 plantations sites within the first two principal components to delimit the Austrian landscape. For prediction of the future climate we used the Max Planck Institute Earth System model (MPI-ESM-LR) under the Rcp45 scenario (Giorgetta et al., 2013), for the period 2041–2060 (**Figure 1**).

### Phenotyping and Genotyping

Phenotyping was conducted on the 21 reforestation sites after excluding individuals with damage and/or bad form. In total, 4,267 trees were measured for height [m] and scored for wood density using the pilodyn penetration method (Cown, 1978). Individual tree position was determined by triangulation. First, a compact block of trees was identified within each reforestation site (denoted as random selections) (Lstibůrek et al., 2015). Second, within each site and based on height measurements, the top-ranking 25% phenotypes were identified as selection candidates (pre-selection) (Lstibůrek et al., 2015). To account for common environment effects, the average of eight direct neighbors was subtracted from the phenotypic observation of a given selected individual (Zobel and Talbert, 2003), and the adjusted values were used in the genetic evaluation. The total number of pre-selection individuals across the 21 reforestation sites is 1,088 (representing 579 and 509 random and top-phenotype selections, respectively). This sample size was optimized to meet three important criteria: 1) achieving comparable genetic response to selection to that of traditional recurrent selection with structured control crosses (i.e., progeny testing) (White et al., 2007), 2) reconstructing pedigree with sufficient accuracy (Marshall et al., 1998; Kalinowski et al., 2007), and 3) satisfying the declared effective population size (i.e., genetic diversity) in the target seed production population (Lstibůrek et al., 2011). The above calculation of sample size also accounted for anticipated pollen contamination, i.e., paternal contributions originating from parents outside the seed orchard (Lstibůrek et al., 2012).

Tissue samples for DNA extraction were collected using a 15-mm hole-punch to obtain cambium cell layers. Samples were immediately dried and stored in silica gel. DNA extraction followed a modified CTAB protocol (Lefort and Douglas, 1999), using app. 100 mg of frozen cambium tissue after grinding in a Mixer Mill MM200 (Retsch). Extracted DNA was fingerprinted using three microsatellite multiplexes accommodating 5 (Ld30, bcLK189, bcLK228, bcLK263, and Ld56), 4 (Ld31, Ld50, bcLK211, bcLK253), and 4 (Ld58, Ld42, Ld101, 4 Ld45) (Wagner et al., 2012). In total, 53 parental and 1,088 offspring trees were genotyped.

### Pedigree Reconstruction

**Figure 2:**
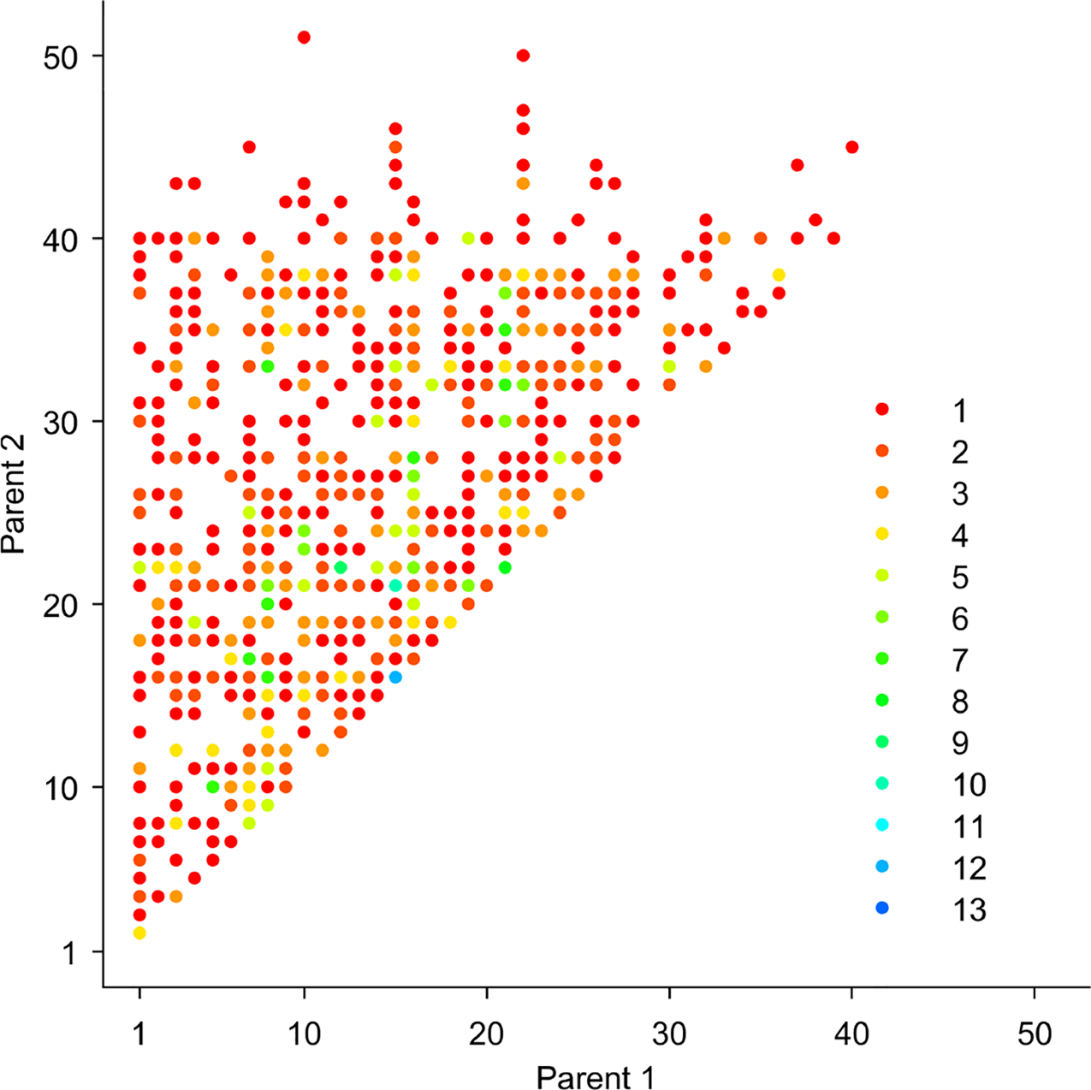
Pedigree reconstruction results showing the formation of full-sib families and their parental combinations and respective family sizes (1-13, reciprocal crosses were grouped) (N = 1,024 offspring).

We performed pedigree reconstruction, yielding 1,024 offspring in 491 full-sib families, representing the largest known forest tree pedigree assembly. The likelihood-based method Cervus (Marshall et al., 1998) was used to reconstruct family relationships (**Figure 2**). Pedigree analysis parameters were: unknown sexes, no assumption for putative maternal contributor, LOD score (natural logarithm of the overall likelihood ratio), and Delta (the difference in LOD between the two most likely candidate parents), reflecting inputted parameters of genotyping errors and incomplete sampling of the parental population. Initial simulation of parentage analysis was processed for 10,000 offspring based on 53 unique genotypes that were considered as candidate parents with six parameter scenarios, including input parameters such as proportion of sampled parental population (0.5, 0.6, 0.7, 0.8, 0.9, 1) and maximal genotyping error rate (0.01, 0.1, 0.01, 0.1, 0.01, 0.1) to assess the parentage assignment robustness. Additional parameters include: minimum number of typed loci of 6, monoecious species with polygamous mating, consideration of selfing, and parentage assignment 99% confidence were kept equal. Only consistent outcomes of family assignment across all scenarios were accepted and used in downstream analysis.

### Statistical Analysis

Pedigree-based genetic analyses were used and variance components, heritabilities, genetic correlations, and individual tree breeding values were estimated/predicted using the bivariate animal model (Henderson, 1984), combining genotyped parental trees and offspring records trees after excluding those with external male parents as follows:

(1) y=Xβ+Za+Yd+Wu+e

where **y** is the vector of observations for the two traits; **X** is the incidence matrix for the fixed effect ***β*** (trait means); **Z** is the corresponding incidence matrix related to random additive genetic effects (breeding values, $\text{a}∼N(0,\sigma_{a}^{2}))$; **Y** is the incidence matrix related to random dominance genetic effects $d∼N(0,\sigma_{d}^{2}))$, while **W** is an incidence matrix for random genotype by environment (or climatic region) interaction $u∼N(0,\sigma_{u}^{2}))$, and the random residual error effects are distributed as $e∼N(0,\sigma_{e}^{2}))$.

The covariance matrix for the random additive genetic effects was modelled using the heterogeneous covariance structure as

(2) $$\sigma_{a}^{2}=\left[\begin{array}{cc} \sigma_{a1}^{2} & \sigma_{a1a2}\\ \sigma_{a2a1} & \sigma_{a2}^{2} \end{array}\right]⊗\text{A}$$

where **A** is the average numerator relationship matrix, *σ_a_*_1_ *_a_*_2_ is the additive covariance between traits 1 and 2, and ⊗ is the Kronecker product operator. A corresponding structure was used for the dominance effects with $\sigma_{a}^{2}$ being replaced by $\sigma_{d}^{2}$ and **A** by **D**, i.e., the dominance genetic relationship matrix. The covariance matrix for the random site effects (genotype by environment interaction) was modelled using a heterogeneous general correlation matrix (equivalent of an unstructured covariance matrix, but with different parametrization) suitable for such a complex correlation structure as

(3) $$\sigma_{u}^{2}=\left[\begin{array}{cc} \sigma_{a1e}^{2} & r_{12}\\ r_{21} & \sigma_{a2e}^{2} \end{array}\right]⊗\text{I}$$

where **I** is the identity matrix.

The random residual error effect was modelled using an unstructured covariance matrix structure as

(4) $$\sigma_{e}^{2}=\left[\begin{array}{cc} \sigma_{e1}^{2} & \sigma_{e1e2}\\ \sigma_{e2e1} & \sigma_{e2}^{2} \end{array}\right]⊗\text{I}$$

where *σ_e_*_1_ *_e_*_2_ is the residual covariance between the two traits. Random effects were assumed to be independent. The above genetic evaluation was conducted in ASReml software (Gilmour et al., 2008).

### Future Parental Population Selection

A selection index was calculated with equal economic weighting on both height and wood density traits. A linear optimum selection model was constructed to maximize the selection response, while meeting the prescribed effective population size in the target seed orchard (Lstibůrek et al., 2015). All selections were unrelated in order to minimize inbreeding depression in seed orchard crop, i.e., future forest plantations. The Optimum-Neighborhood Algorithm was implemented to promote panmixia within the new orchard (Chaloupková et al., 2016).

## Results

**Figure 3:**
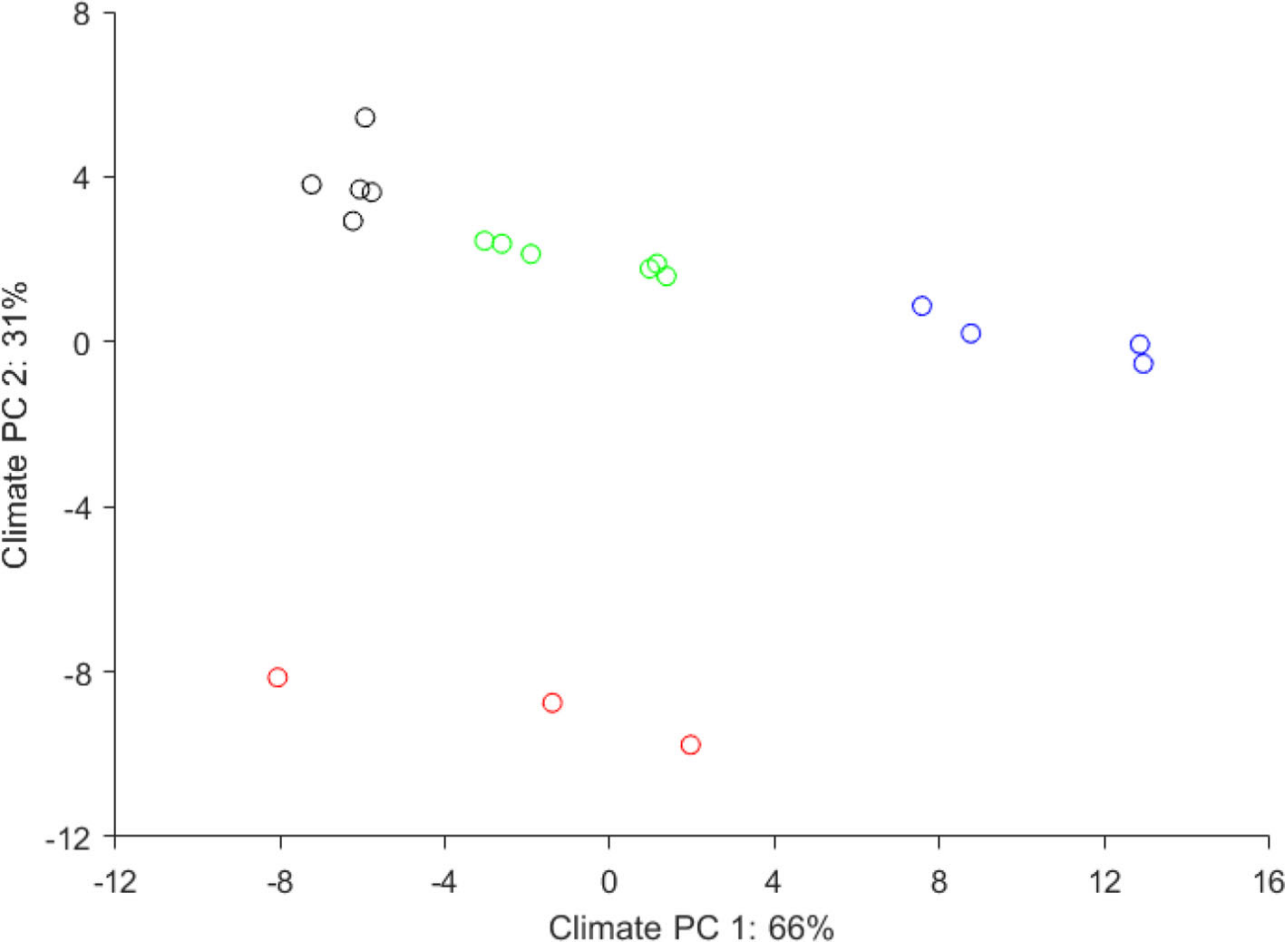
Climatic distribution of the 21 European larch reforestation installations grouped following principal component analysis of climatic parameters. The four climatic groups represented: 1) continental Pannonian climate (black), 2) temperate climate with Atlantic influence (red), 3) a temperate climate with continental influence (green) and, 4) a mountainous climate (blue). These were used as climatic categories to test the presence or absence of genotype-climate interactions.

**Figure 4:**
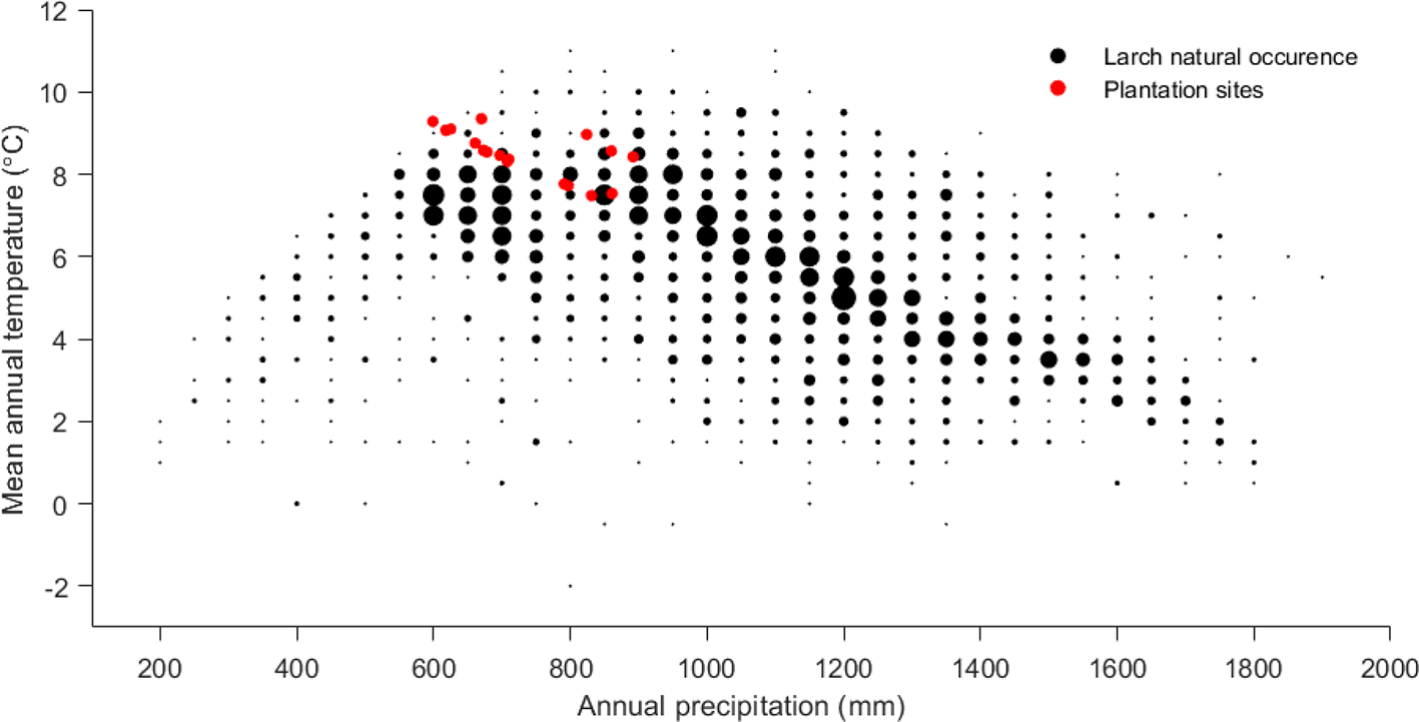
Climate (precipitation and temperature) of the European larch reforestation installations in comparison to species natural occurrence demonstrating that our test sites are close to the warmer border of the species range.

The wide climatic gradient was confirmed by PCA of the plantation site climate, which resulted in four distinct climatic groups (**Figure 3**), thus extending the testing beyond the confinement of a defined ecological testing target. Furthermore, the reforestation installations were grouped at the “warmer end” of the species distribution (**Figure 4**), thus offering stronger environmental testing conditions (i.e., additional “ecological tension”).

Pedigree reconstruction assembled 491 full-sib families, representing 35% of the possible 53-parent half diallel (**Figure 2**). This represents the largest known forest tree pedigree assembly. There was gametic contribution from the entire orchard's parental population, resulting in 1,088 offspring available for the quantitative genetics analyses. It is interesting to note that the gene flow from outside the orchard accounted for 8.4% and 3.4% of the observed matings in the random and pre-selection samples, respectively, meeting theoretical expectations (Lstibůrek et al., 2012). Additionally, offspring resulting from self-pollination was not detected.

Variance components were estimated from the multi-site bivariate mixed linear model. The final model with fixed site and random pedigree effects resulted in heritability (*h* ^2^) estimates of 0.25 (SE = 0.065) and 0.30 (SE = 0.072) for height and wood density, respectively, corresponding to reported estimates from Larix species “structured” testing trials (Pâques, 2004; Ratcliffe et al., 2014). Both traits produced non-significant dominance interaction, thus simplifying the model, and the expected family performance was estimated by the mean additive genetic value of the two respective parents. Negative but negligible genetic correlation between height and wood density was observed (-0.04 ± 0.20), corresponding to the known general trend in most conifers (Zobel and Jett, 2012).

No significant genetic variation by environment (site) interaction was observed across the 21 studied sites, leading us to conclude that individuals' additive genetic values are indicative of their general performance across the range of studied sites and that the studied population consists of generalists that performed well under wide site and climatic conditions and further demonstrating that the selected individuals would form appropriate seed production population for planting over a wide climate regime range including potential future conditions (**Figure 1**). These conclusions are supported by the fact that respective parents/families have been tested across a broad range of environments, and selections have been identified across all sites.

Following the genetic evaluation, we selected 25 unrelated offspring individuals with which to establish the new seed production population with improved climate change adaptability and productivity; thus, the phenotyping and genotyping effort were sufficient in capturing the target effective population size. Selected individuals yielded genetic responses of 1.1 and 0.7 standard deviations for fitness and productivity attributes, respectively. Scions collected from selected offspring were grafted onto rootstocks, and the second-generation seed orchard was established (the advanced generation seed production population).

## Discussion

In this manuscript, we developed and validated a novel large-scale *in situ* forest gene resource management scheme to identify productive and climatically adapted individuals originating from an Austrian European larch seed production population (seed orchard), utilizing traditional reforestation installations planted widely across the landscape and spanning three decades. Our thesis is based on the expectation that thriving individuals within these installations have been spatially and temporary challenged and thus have effectively dealt with the negative impacts of climate change. In conventional selective breeding programs, a structured pedigree is produced from controlled pollinations following specific mating designs (White et al., 2007), and evaluated in replicated test sites within defined ecological boundaries (Hanewinkel et al., 2013), a prerequisite for effective genetic evaluation and selection. Our approach is anchored on meeting two conditions, namely, the successful assembly of a “structured pedigree” from seed orchard offspring produced under natural pollination (El-Kassaby and Lstibůrek, 2009), and whether progeny evaluation can be conducted within reforestation installations (Lstibůrek et al., 2015) rather than progeny test sites. Meeting these two conditions effectively bypasses the conventional breeding and testing phases used in recurrent selection strategies, thus accelerating and simplifying the selection process. Given that reforestation sites are usually employed across more and a wider range of site and climate conditions than traditional genetic trials, our approach also allows predicting potential application regions for the improved forest seeds. However, for the full Alpine range of larch and for a climate warmer than observed today, the application might be restricted, as we could expect GxE interaction outside of the current conditions (Koralewski et al., 2015).

This is the first proof of the “Breeding without Breeding, BwB” concept (El-Kassaby and Lstibůrek, 2009) in a large operational forestry program. Our results are in agreement with the respective theoretical expectations published earlier. First, phenotypic preselection provided sufficient distribution of candidates meeting the prescribed effective population size of the new seed orchard, which is in agreement to Lstibůrek et al. (2011). Second, phenotypic preselection was efficient at reducing the contamination rate among the parents of the genotyped subset of offspring, which corresponds to both theoretical expectations (Lstibůrek et al., 2012) as well as the actual findings in Scots pine (Korecký et al., 2014). As noted earlier, actual heritabilities, genetic correlations, and respective standard errors are within the range of conventional breeding programs, facilitating genetic gains that were also in agreement to computer simulations and deterministic expectations (Lstibůrek et al., 2015). Further, we observed the beneficial effect of the increased size of the candidate population (i.e., an increase in selection intensity), which further boosted the genetic response of selection (Lstibůrek et al., 2017) beyond the levels of conventional breeding programs. We can therefore conclude that BwB strategies provide an effective and economically feasible method to breed outcrossing forest tree species. We therefore forecast the utility of BwB methods in operational forestry as they facilitate full-scale landscape gene-resource management of forest trees.

In line with the discussion in Lstibůrek et al. (2017), we advocate the utility of BwB approaches (such as the current study) for the following reasons. (1) Absence of full-sib crosses, as the method relies on natural pollination in breeding arboretums (seed orchards). (2) Absence of progeny trials. Genetic testing can be performed within commercial forest stands. (3) Genetic evaluation thus takes place on a landscape level, emphasizing adaptive traits and their respective interaction with environmental conditions. (4) Strategies are open to NGS platforms. One can replace the BLUP based evaluation (as implemented here) with the genomic alternative (e.g., GBLUP) with all the added benefits of extracting additional genetic parameters (El-Dien et al., 2016). When considering these alternatives, breeders may compare theoretical gain efficiencies between BLUP and GBLUP approaches (Stejskal et al., 2018). At the same time, operational implementation could still remain identical to the current study.

In summary, the approach presented here is a flexible and dynamic gene-resource management scheme that is not encumbered by the predetermined fixed ecological zonation, commonly implemented for forest trees. The reforestation installations permitted effective and rigorous genetic evaluation over numerous sites with varying ecological diversity (geographic distribution) and extended the testing timeframe, thus speeding the selection of adapted individuals and matching them with the most appropriate planting location. The approach is simple and cost-efficient, enabling improvement and conservation of commercial and non-commercial species under rapidly changing environmental conditions.

## Data Availability Statement

The datasets analyzed for this study can be found in https://github.com/mlstiburek/European-larch.

## Author Contributions

ML, SS, YE-K, and TG conceived the project and designed the study. ML estimated sample sizes. SS, HK, and TG carried out the genotyping. PŠ coordinated the fieldwork. JK conducted the pedigree reconstruction. GH, JS, and ML ran statistical analyses. ML, SS, TG, YE-K, JK, and JS contributed to writing the manuscript.

## Funding

This research was funded by OP RDE grant Extemit-K, No. CZ.02.1.01/0.0/0.0/15003/0000433 (ML), the Austrian Research Promotion Agency (FFG) and the Cooperation Platform Forst Holz Papier (FHP) and LIECO nurseries and the Austrian Federal Forests (ÖBf) (SS, PŠ, HK, and TG), the Natural Sciences and Engineering Research Council of Canada (NSERC) Discovery grant and the Johnson's Family Forest Biotechnology Endowment (YE-K), Camcore, Department of Forestry and Environmental Resources, NC State University (GH).

## Conflict of Interest

The authors declare that the research was conducted in the absence of any commercial or financial relationships that could be construed as a potential conflict of interest.
